# Early signaling events as vital regulators of enzymatic browning in fresh-cut fruits and vegetables: a mini-review

**DOI:** 10.3389/fpls.2026.1920175

**Published:** 2026-10-07

**Authors:** Zhaorui Zhu, Xiao Yuan, Siqi Xu, Zhaoxia Zhan, Jinming He, Bin Wang

**Affiliations:** 1 College of Biology and Agriculture, Shaoguan University, Shaoguan, China; 2 Guangdong Provincial Key Laboratory of Utilization and Conservation of Food and Medicinal Resources in Northern Region, Shaoguan University, Shaoguan, China; 3 Guangdong Provincial Engineering and Technology Research Center of Special Fruit and Vegetables in Northern Region, Shaoguan University, Shaoguan, China

**Keywords:** enzymatic browning, fresh-cutting, fruit and vegetable, lipid metabolism, signal transduction

## Abstract

Unlike intact fruits and vegetables, fresh-cut produce undergoes minimal processing operations that render it highly susceptible to quality deterioration. Among the adverse quality issues, surface browning represents a primary obstacle curtailing the post-processing shelf life and marketability of minimally processed commodities. While classical paradigms regarding fresh-cut browning predominantly center on downstream, polyphenol oxidase-mediated enzymatic reactions, this mini-review highlights the critical importance of early signaling events. These include wounding signal perception, ROS burst, Ca^2+^ signal transduction, rapid phytohormone response, and their subsequent crosstalk during the imitation of browning. Shifting the research paradigm toward a comprehensive upstream regulatory network will pave the way for engineering next-generation postharvest browning interventions.

## 1 Introduction

Fresh-cut fruits and vegetables are minimally processed products, prepared for immediate consumption. These products undergo a sequence of operations, including washing, peeling, slicing, dicing, and packaging, followed by storage under low temperatures (Yuan et al., 2026a). Consumer appeal of fresh-cut produce stems from its perceived freshness, convenience, enhanced nutritional and flavor retention, and high edible rate (Ma et al., 2017). Nevertheless, despite the application of cold storage, fresh-cut produce exhibits accelerated deterioration during post-processing storage. This phenomenon is predominantly attributed to the physical injuries inflicted during the cutting process, manifesting as tissue softening, cut-surface browning, diminished nutritional quality, development of undesirable flavors, and microbial spoilage (Kim and Cheigh, 2022; Wang et al., 2022; Nyamende et al., 2023; Rasines et al., 2024).

Among these adverse quality issues, browning is of paramount concern, as the visual appearance of fresh-cut produce directly dictates its perceived freshness and subsequent consumer purchase intentions (Yuan et al., 2025a). Although numerous review articles have comprehensively summarized the mechanisms and control strategies of enzymatic browning (Arnold and Gramza-Michałowska, 2022; Zhang et al., 2022a; Sui et al., 2023; Liu et al., 2025a; Sun et al., 2026), they remain predominantly centered on classical polyphenol oxidase (PPO)-mediated enzymatic reactions. However, emerging evidence over the past decade indicates that these downstream reactions do not occur in isolation but are governed by upstream, key triggers. Specifically, early signaling events, such as rapid reactive oxygen species (ROS) bursts, Ca^2+^ signal transduction, and phytohormone homeostatic shifts, may act as vital regulators that orchestrate both the onset and severity of enzymatic browning.

Therefore, it is imperative to update our mechanistic insights by shifting the focus from downstream output to upstream regulation. To bridge this knowledge gap, this mini-review provides a holistic, systemic framework that integrates these early signaling pathways into a unified regulatory scheme, thereby paving the way for developing next-generation postharvest browning interventions.

## 2 The activities of PPO or POD frequently misalign with fresh-cut browning

It has been widely established that enzymatic reactions dominate the browning process in fresh-cut produce. In these reactions, oxidative enzymes catalyze the oxidation of phenolic substrates into quinones in the presence of molecular oxygen or hydrogen peroxide (H_2_O_2_) (Tilley et al., 2023). These highly reactive quinones subsequently undergo non-enzymatic polymerization, culminating in the accumulation of dark melanin-like pigments that manifest as browning (Wang et al., 2024). Consequently, classical paradigms dictate that both oxidative enzymes and phenolic compounds constitute the indispensable, rate-limiting components of the enzymatic browning reaction.

These oxidative enzymes, predominantly comprising PPO and peroxidase (POD), are ubiquitously distributed within the plant kingdom (Vámos-Vigyázó, 1981; Masiala et al., 2024). Traditionally, PPO represents the primary enzyme implicated in browning and is systematically classified into two types based on substrate specificity: monophenol monooxygenase (EC 1.14.18.1, tyrosinase and cresolase) and diphenol oxidase (EC 1.10.3.1, catechol oxidase) (Moon et al., 2020). Conversely, POD contributes to oxidative browning by driving the oxidation of endogenous phenols and monolignols by utilizing H_2_O_2_ as a co-substrate (Senthilkumar et al., 2020). The operational distinction between these two oxidases lies in their co-substrate dependencies: PPO-driven enzymatic browning requires molecular oxygen, whereas POD-mediated reactions are driven by H_2_O_2_ availability (Yuan et al., 2026a). Furthermore, POD possesses the catalytic capability to polymerize monolignols into lignin, thereby mediating structural lignification (Zhang et al., 2022b), a process intimately correlated with tissue discoloration in fresh-cut produce.

Given this established biochemistry, one would logically expect browning severity to tightly couple with enzyme abundance. However, growing evidence reveals a striking misalignment between PPO or POD activity profiles and actual browning progression. For example, during the development of under-skin browning in mango fruit, total phenolic content increases along with browning, whereas PPO and POD activities substantially decrease (Li et al., 2021). Similarly, the ‘Topaz’ apple cultivar remains highly sensitive to browning despite exhibiting the lowest baseline PPO and POD activities among 14 compared cultivars (Serra et al., 2021). Another compelling paradox occurs in fresh-cut taro, where the application of peppermint extracts significantly attenuates browning development while concurrently enhancing PPO and POD activities (Yuan et al., 2023). These contradictory reports unambiguously demonstrate that the absolute activities of PPO or POD frequently misalign with phenotypic browning, suggesting that alternative, highly complex upstream regulatory mechanisms may dictate the ultimate browning progression.

## 3 Possible perception of mechanical wounding in fresh produce

Mechanical wounding during fresh-cut processing immediately disrupts cell wall integrity and plasma membrane continuity, triggering an instantaneous alarm system in remaining intact cells (Liu et al., 2025b). This profound cellular disruption serves as the primary driver of wounding signals, distinguishing fresh-cut browning from the physiological browning observed in intact fruits. The primary perception of this wounding stress involves a sophisticated synergy between physical mechanosensors and chemical receptor-mediated recognition of damage-associated molecular patterns (DAMPs in plants).

### 3.1 Mechanosensitive channels and membrane receptors

Mechanical shearing at the cutting interface induces abrupt turgor pressure fluctuations and cell wall deformations that serve as the primary physical stimuli (Hamant and Haswell, 2017). Plants perceive these mechanical forces *via* specialized plasma membrane-embedded mechanosensitive ion channels. Among these, mechanosensitive (MS) channels of small conductance-like (MSL) proteins function as the initial force-gates (Basu and Haswell, 2020). Upon mechanical wounding, the consequent stretching of the lipid bilayer directly gates these MS channels, specifically the MSL families, driving an instantaneous influx of extracellular cations, predominantly Ca^2+^ (Fichman and Mittler, 2020; Yu et al., 2024).

Furthermore, recent reports indicate that plant Piezo channels are involved in mechanical stress perception in model systems like *Arabidopsis thaliana* (Peng et al., 2025). However, direct electrophysiological or genetic validation of Piezo channels as wounding sensors in horticultural crops has not yet been reported. Moreover, plant Piezo channels are primarily localized to the vacuolar membrane rather than the plasma membrane (Radin et al., 2021), inferring their role in horticultural produce might be restricted to vacuolar turgor regulation rather than sensing the initial cell-surface shear stress.

Concurrently, studies in model plants have established that wall-associated kinases (WAKs) and receptor-like kinases (RLKs), most notably the Feronia (FER) CrRLK1L receptor, actively monitor pectin integrity (Feng et al., 2018). When the pectin matrix is breached by shearing, the localized alteration in pectin cross-linking dynamically reconfigures FER-mediated surveillance signaling, translating wound-induced mechanical strain into intracellular cascades that regulate downstream acclamatory responses (Shen et al., 2025). Within model systems, this pathway has been shown to modulate the expression of pivotal wound-responsive transcription factors, such as ERF115 (Canher et al., 2022). Nevertheless, this FER-dependent cell wall integrity surveillance pathway likely displays substantial commodity-specific divergence in fresh-cut produce. In parenchymatous tissues characterized by abundant, highly methylesterified pectin, such as fresh-cut apples, mechanical tearing rapidly liberates oligogalacturonide (OG) elicitors into the apoplast to ignite downstream phenylpropanoid synthesis (Chen et al., 2025). Conversely, in highly lignified tissues like fresh-cut bamboo shoots, the dense secondary cell wall matrix and distinct pectin cross-linking profiles may significantly attenuate the sensitivity of the FER complex to mechanical breaches (Xie et al., 2026). This structural interference potentially decouples the sensory interface from downstream transcriptional activation, presenting a major unresolved question in postharvest mechanobiology.

### 3.2 DAMPs as wounding signals

Upon mechanical wounding, damaged plant cells liberate a diverse array of endogenous molecules that function as vital danger signals, collectively termed damage-associated molecular patterns (Tanaka et al., 2014). Among these, intracellular ATP is passively released into the apoplast following cellular rupture, where it acts as a primary DAMP (Tanaka et al., 2010; Cao et al., 2014). This extracellular ATP (eATP) is specifically perceived by the plasma membrane-bound purinergic receptor DORN1/P2K1, rapidly triggering a downstream oxidative burst and mitogen-activated protein kinase (MAPK) phosphorylation cascades (Choi et al., 2014). Concurrently, cell wall-derived pectin fragments, specifically OGs, have traditionally been thought to bind to WAKs to further amplify the defense response in plants (Ferrari et al., 2013). However, recent genetic evidence utilizing an *Arabidopsis* mutant lacking all five *WAK* genes has shown that WAKs are actually dispensable for OG-induced signaling and immunity (Herold et al., 2025), questioning their proposed role as *bona fide* OG receptors. Synergizing with initial physical stimuli, these danger signals are postulated to orchestrate a robust, localized stress memory at the cutting interface to prime the tissue for subsequent metabolic shifts and enzymatic browning.

Despite the conceptual elegance of this DAMP-mediated alarm network, its direct involvement in fresh-cut browning remains largely inferred from broader plant stress biology rather than conclusively demonstrated. While eATP-DORN1 signaling is standard in vegetative defense (Chen et al., 2017), the expression levels and functional binding affinities of P2K1-type receptors vary widely among different crop commodities and ripening stages. In senescing or overripe fruits, the background levels of apoplastic eATP are already elevated due to physiological cell breakdown, which is hypothesized to desensitize the DORN1 receptor and render the tissue unresponsive to further cutting-induced eATP spikes (Shan et al., 2020).

Furthermore, competing hypotheses persist regarding the role of OG-WAK interaction in wounding. One school of thought argues that OG-WAK binding induces massive ROS generation and upregulates phenylalanine ammonia-lyase (PAL) transcription (Denoux et al., 2008). An alternative, competing hypothesis suggests that in specific commodities, the OG-mediated activation of WAKs primarily drives structural wound healing and suberization, which serves to seal the wound and ultimately restrict long-term enzymatic oxidation (Lulai and Suttle, 2009). Therefore, rather than forming a uniform, integrated network, the DAMP signaling landscape in fresh-cut produce may be highly conditional, governed by the baseline physiological status and specific matrix attributes of the individual commodity.

## 4 Key early signaling cascades regulating enzymatic browning in fresh-cut produce

Following the perception of mechanical trauma at the cutting interface, intracellular alarm signals are rapidly amplified and propagated through a highly interconnected network of secondary messengers. This early signaling cascade operates within seconds to minutes post-wounding, acting as the critical molecular conduit that converts physical shearing forces into systemic biochemical responses. The orchestration of enzymatic browning relies heavily on the synergistic crosstalk among three core intracellular cascades: ROS burst, Ca^2+^ wave propagation, and rapid phytohormone response.

### 4.1 ROS burst

Mechanical wounding during the fresh-cut processing of fruits and vegetables immediately triggers an abrupt and localized accumulation of ROS, widely designated as the ROS burst. This primary event occurs within seconds to minutes post-cutting, driven predominantly by the rapid activation of plasma membrane-bound nicotinamide adenine dinucleotide phosphate (NADPH) oxidases, structurally identified as respiratory burst oxidase homologs (Rboh) (Shi et al., 2024a). These enzymes catalyze the transfer of electrons from cytoplasmic NADPH to extracellular oxygen, generating superoxide radicals, which are subsequently dismutated into more stable and membrane-permeable H_2_O_2_ (Li et al., 2023). While chronic accumulation of ROS inevitably induces lipid peroxidation and compromises membrane structural integrity, this initial, acute influx of localized H_2_O_2_ acts strictly as a vital second messenger (Liu et al., 2021).

Nevertheless, whether the ROS burst serves as a general switch for PPO activation across all fresh-cut commodities remains a subject of ongoing debate. While direct genetic and biochemical lines of evidence have confirmed Rboh-driven ROS signaling and subsequent browning or wound healing in solanaceous crops like potatoes (Wang et al., 2023), in highly acidic fruits (e.g., citrus or green apples), the localized tissue pH drop and high-capacity pools of non-enzymatic antioxidants (such as ascorbic acid) frequently mask or decouple the downstream regulatory effects of the initial oxidative shock (Moon et al., 2020). Specifically, because PPOs typically exhibit optimal activity at pH 5.0-7.0 and are strongly inactivated below pH 3.0, the profound endogenous acidity and active ascorbate-glutathione cycle in these fruits act as a biochemical buffer that suppresses early melanin formation, irrespective of the upstream wound-induced Rboh activation.

### 4.2 Ca^2+^ signal transduction

Simultaneously with the ROS burst, the physical shock of mechanical cutting induces an instantaneous influx of extracellular Ca^2+^ into the cytosol, serving as a critical upstream biophysical signal (Shi et al., 2024b). This rapid influx is mediated through the activation of mechanosensitive ion channels and cyclic nucleotide-gated channels situated on the plasma membrane, which open in response to the sudden alteration in cellular turgor pressure (Feng et al., 2022). The resulting transient spike in intracellular Ca^2+^ concentration is immediately decoded by specialized, high-affinity intracellular calcium sensors (Gao et al., 2019).

Critically, a major unresolved question in postharvest biology is how fresh-cut tissues distinguish generalized mechanical touch from destructive shearing forces. While specific downstream Ca^2+^ sensors like CDPKs and CaMs are linked to stress responses in vegetative systems, direct experimental verification in fruit and vegetable browning remains scarce. For example, while exogenous calcium interventions suppress enzymatic browning in fresh-cut luffa (*Luffa acutangula*), these studies evaluate downstream antioxidant enzyme profiles (e.g., SOD, CAT) rather than tracking upstream sensor decoding (Feng et al., 2022). Direct evidence for CDPK or CaM bridging trauma and PPO activation is mostly restricted to non-horticultural model systems (Gao et al., 2019).

### 4.3 Rapid response of phytohormones

Following the initial biophysical and chemical shifts, endogenous phytohormones function as systemic mediators that coordinate the spatial progression of the wound response across the fresh-cut tissue. Mechanical stress induces the rapid biosynthesis of jasmonic acid (JA), salicylic acid (SA), and ethylene (ET) within minutes to hours after cutting (Fugate et al., 2023). Among these, JA and ET serve as the two primary wound-responsive phytohormones in plants. Wounding upregulates the lipoxygenase (LOX)-mediated oxylipin pathway, triggering a massive accumulation of JA at the cut site, which frequently acts as the primary hormonal driver of wound-induced secondary metabolism (Upadhyay and Mattoo, 2018).

However, the functional hierarchy and spatial distribution of these hormonal cascades exhibit immense commodity-specific variation (Toivonen and Brummell, 2008). For instance, while the JA-driven upregulation of *PAL* and *PPO* transcription is well established in fresh-cut leafy and petiole tissues such as lettuce (*Lactuca sativa*) and celery (*Apium graveolens*) (Saltveit, 2000; Cantos et al., 2001), the hormonal regulation of browning in other commodities operates through an intricate, species-dependent network. In these cases, ethylene or alternative hormonal signaling pathways can emerge as the primary, context-dependent drivers of tissue discoloration, a phenomenon dictated entirely by the specific crop species, developmental stage, and tissue type (Guo et al., 2026).

### 4.4 Potential crosstalk between early signals upon wounding

The individual signaling events do not function in isolation; rather, they may form a complex, highly integrated crosstalk network that orchestrates the severity of enzymatic browning. The initial ROS burst directly shapes Ca^2+^ dynamics, as the localized accumulation of H_2_O_2_ oxidatively modulates and activates ROS-responsive plasma membrane Ca^2+^-permeable channels, thereby amplifying the cytosolic calcium influx (Pottosin et al., 2014). In a reciprocal positive-feedback loop, the activated Ca^2+^/CaM complex and specific CDPKs directly bind to and phosphorylate the Rboh protein, enhancing its NADPH oxidase activity and sustaining the ROS signaling threshold in plants (Kobayashi et al., 2007; Gao et al., 2019). Concurrently, this convergent ROS-Ca^2+^ signaling hub may serve as an indispensable upstream trigger for the rapid biosynthesis of JA and ET by upregulating key biosynthetic transcripts, LOX and 1-aminocyclopropane-1-carboxylic acid oxidase (Howe et al., 2018; Fugate et al., 2023). Conversely, endogenous gasotransmitters, such as nitric oxide and hydrogen sulfide, act as critical antagonistic modulators within this post-wounding network to buffer excessive browning cascades (Qiao et al., 2025; Zhao et al., 2026).

While this integrated regulatory network offers a compelling conceptual framework, it inadvertently conflates distinct mechanisms that may co-exist only under specific physiological conditions or within specific plant families. For example, the CDPK-mediated phosphorylation of Rboh is supported by robust direct evidence in solanaceous species (Kobayashi et al., 2007); however, its active operation during the enzymatic browning of non-solanaceous fresh-cut fruits remains an association largely inferred from independent abiotic stress studies rather than direct postharvest processing contexts (Gao et al., 2019). Future reviews and empirical models should carefully delineate these broadly established core pathways in plants from tentative, commodity-specific observations to avoid over-generalizing the molecular architecture of fresh-cut browning.

## 5 Downstream activation of enzymatic browning by early signal events

The transduction of upstream signals into phenotypic browning may rely on MAPK cascades. In fresh-cut tissues, the mechanical stress-induced ROS burst and Ca^2+^ flux activate specific MAPKs, which sequentially phosphorylate downstream transcriptional regulators rather than functioning as generalized activators. For instance, transcriptomic and metabolomics analyses in fresh-cut sweetpotato (*Ipomoea batatas*) demonstrate that physical cutting sharply triggers the MAPK signaling pathway, initiating immediate upstream transcriptional reprogramming, including specific MYBs and WRKYs, to drive downstream phenolic accumulation and enzymatic browning (Liu et al., 2026). Similarly, in potato (*Solanum tuberosum*) tubers, mechanical trauma and secondary oxidative stresses have been directly linked to the rapid activation of MAPK clades that interface with phenylpropanoid biosynthesis to govern the initial postharvest stress response (Zheng et al., 2022).

Once activated by upstream signaling networks, specific TFs orchestrate enzymatic browning by binding directly to the promoter regions of browning-related genes such as *PPO* and *PAL* to activate their transcription. This transcriptional step is governed by distinct regulatory modes rather than a uniform activation pattern, exhibiting high commodity and cultivar specificity. In red-fleshed apple (*Malus domestica*) cultivars, transcriptomic and metabolomics profiles indicate that while downstream pathways involve PPO dynamics, the early browning stage is predominantly characterized by the sharp expression of context-specific *WRKY* and *AP2/ERF* families, which operate alongside the classical MYB regulatory cascades (Zuo et al., 2021). Direct molecular evidence of transcriptional activation has been robustly characterized in horticultural commodities; for example, the AcWRKY65 has been explicitly demonstrated to bind directly to the promoter region of *AcPPO*, functioning as a potent positive regulator that activates its expression to drive postharvest browning symptoms (Zhang et al., 2027). Conversely, specific transcriptional repressors are deactivated during trauma; in peach (*Prunus persica*), under normal homeostatic conditions, a R2R3-MYB TF, PpMYB73, binds directly to the promoter of *PpPPO1* to suppress its transcription (Zhao et al., 2026).

The final physical trigger for enzymatic browning may be the loss of subcellular compartmentalization, a process driven biophysically by early ROS accumulation and membrane lipid deterioration (Yuan et al., 2026b). Under normal physiological conditions, PPOs are physically sequestered within plastids, while their phenolic substrates remain isolated safely inside the vacuole (Toivonen and Brummell, 2008). The localized ROS burst induced by fresh-cutting directly or indirectly stimulates the activities of key membrane-degrading enzymes, principally phospholipase D (PLD) and LOX, which systematically accelerate the degradation of structural phospholipids (Zhu et al., 2023). This enzymatic attack leads to severe lipid peroxidation of cellular membrane, causing progressive micro-ruptures and loss of selective membrane permeability (Yuan et al., 2025b). Once this structural compartmentalization collapses, the phenolic substrates and PPO are liberated into the ruptured cellular matrix and/or apoplastic space, coming into direct contact. This interaction drives the immediate, oxygen-dependent oxidation of phenols into highly reactive *o*-quinones.

While the sequential cascade from upstream trauma to physical membrane collapse represents a biologically plausible framework for enzymatic browning, the intervening transcriptional wiring should not be over-generalized into a single, omnipotent model. Competing evidence suggests that the downstream gene regulatory networks operate with immense evolutionary divergence across plant families. For instance, while direct physical interaction assays have confirmed the positive trans-activation of AcWRKY65 in tropical fruits (Zhang et al., 2027) and the active trans-repression of PpMYB73 in temperate prunus species (Zhao et al., 2026), there remains a severe empirical gap when extrapolating these specific molecular brakes to other fresh-cut commodities like apples (Zuo et al., 2021) or leafy vegetables (Liu et al., 2026). Presenting these scattered, independent observations as a fully integrated grid risks conflating broadly established biophysical phenomena with tentative, commodity-specific anomalies. Furthermore, current conceptual models face clear methodological limitations, as traditional multi-omics tools lack the real-time, single-cell spatiotemporal resolution required to definitively map whether early transcript accumulation is directly driven by specific upstream MAPK modules or is merely a secondary byproduct of broad mechanical shearing.

## 6 Conclusion and perspective

### 6.1 Conclusion

This mini-review evaluates the pivotal role of early upstream signaling cascades in governing the initiation of enzymatic browning in fresh-cut produce. We highlight that enzymatic browning in fresh-cut produce is not merely a passive, wound-induced chemical reaction, but a complex, conditional physiological process orchestrated by early upstream signaling networks. To encapsulate these pathways, a schematic model integrating early signaling events with downstream enzymatic cascades and biochemical reactions is proposed (**Figure 1**). Mechanistically, the immediate biophysical shock of mechanical cutting triggers transient cytosolic Ca^2+^ influxes alongside a rapid ROS burst, which collectively function as the primary cellular alarm system. These secondary messengers converge into intracellular relay systems, most notably MAPK phosphorylation cascades, to drive the biosynthesis and reciprocal crosstalk of wound-responsive phytohormones. Ultimately, this intricate signaling web converts transient stress cues into permanent phenotypic alterations *via* two divergent downstream pathways: the transcriptional activation or de-repression of browning-related genes, and the biophysical degradation of structural phospholipids mediated by lipases, which drives the collapse of sub-cellular compartmentalization. Crucially, as cautioned throughout this review, researchers should avoid over-generalizing these pathways into a single regulatory blueprint, given that the functional hierarchy and spatial dynamics of these early signals display profound species, tissue, and cultivar specificity.

**Figure 1:**
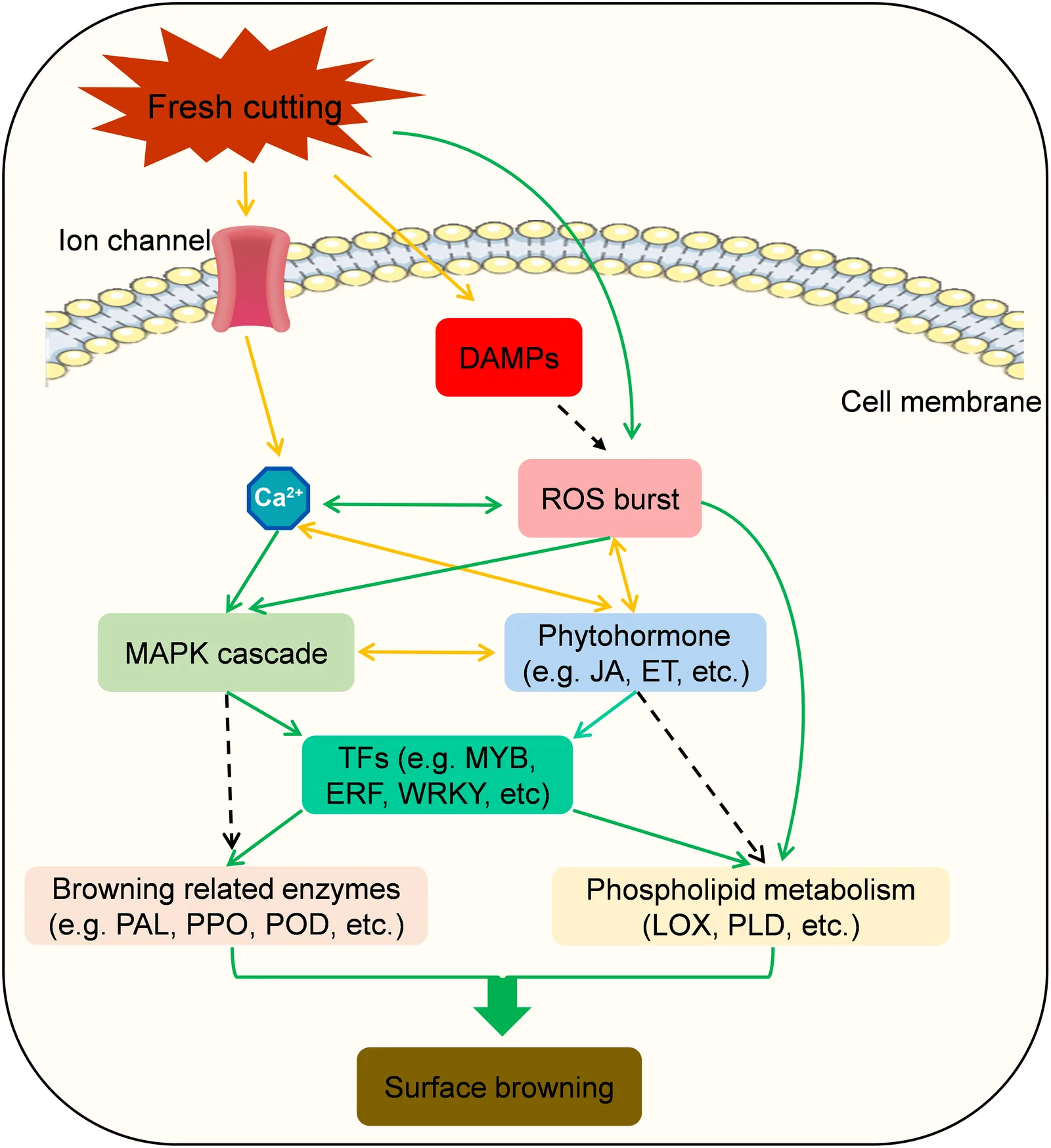
An integrated schematic network of early signaling events regulating enzymatic browning in fresh-cut produce. The evidence hierarchy is graphically represented as follows: **(a)** solid green lines indicate mechanisms directly supported by experimental evidence in fresh-cut browning; **(b)** solid yellow lines indicate mechanisms established in general plant wound, stress, or mechanosensing biology (e.g., in model systems like Arabidopsis) but extrapolated to fresh-cut tissues; and **(c)** dashed lines indicate tentative or commodity-specific relationships. Parts of the figure were adapted from Servier Medical Art, licensed under CC BY 3.0.

### 6.2 Future perspective

To address current empirical gaps and facilitate the transition from conceptual modeling to targeted postharvest preservation, several critical avenues should be prioritized in future research:

1. More studies are needed to elucidate how plant cells decode distinct spatiotemporal Ca^2+^ signatures or ROS oscillations to precisely differentiate non-destructive mechanical touch from fresh-cut shearing forces.
2. Advanced real-time bio-imaging modalities, such as high-resolution fluorescent biosensors or high-speed confocal microscopy are required to map the exact flux of messengers within the initial seconds of physical trauma, while spatial transcriptomics should be deployed to resolve the cellular heterogeneity between injured interface cells and neighboring intact tissues.
3. Functional validation of tentative, commodity-specific molecular regulators *via* gene editing technologies, such as CRISPR/Cas9, in horticultural crops is critical to reducing the current reliance on inferences extrapolated from model vegetative systems.
4. The translation of upstream signaling insights into commercial postharvest practices provides a powerful catalyst to accelerate innovative “priming” technologies (e.g., precise micro-dosing of exogenous gasotransmitters or volatile organic compounds) designed to intercept or reshape early alarm signals before the irreversible loss of cellular compartmentalization.

Ultimately, shifting the postharvest paradigm from a traditional, PPO-centric view to an upstream, signal-focused perspective will pave the way for the development of precise, fine-tuned interventions to mitigate fresh-cut browning.
